# Synthesis of Al_2_Ca Dispersoids by Powder Metallurgy Using a Mg–Al Alloy and CaO Particles

**DOI:** 10.3390/ma10070716

**Published:** 2017-06-28

**Authors:** Junji Fujita, Junko Umeda, Katsuyoshi Kondoh

**Affiliations:** 1 HI-LEX CORPORATION, 1-12-28 Sakaemachi, Takarazuka-City, Hyogo 665-0845, Japan; 2 Graduate School of Engineering, Osaka University, 2-1 Yamadaoka, Suita, Osaka 565-0871, Japan; 3 Joining and Welding Research Institute, Osaka University, 11-1 Mihogaoka, Ibaragi, Osaka 567-0047, Japan; umedaj@jwri.osaka-u.ac.jp (J.U.); kondoh@jwri.osaka-u.ac.jp (K.K.)

**Keywords:** magnesium alloys, powder metallurgy, composite materials, microstructure, phase transformation, transmission electron microscopy

## Abstract

The elemental mixture of Mg-6 wt %Al-1 wt %Zn-0.3 wt %Mn (AZ61B) alloy powder and CaO particles was consolidated by an equal-channel angular bulk mechanical alloying (ECABMA) process to form a composite precursor. Subsequently, the precursor was subjected to a heat treatment to synthesize fine Al_2_Ca particles via a solid-state reaction between the Mg–Al matrix and CaO additives. Scanning electron microscopy-energy-dispersive spectroscopy (SEM-EDS) and electron probe micro-analysis on the precursor indicated that 4.7-at % Al atoms formed a supersaturated solid solution in the α-Mg matrix. Transmission electron microscopy-EDS and X-ray diffraction analyses on the AZ61B composite precursor with 10-vol % CaO particles obtained by heat treatment confirmed that CaO additives were thermally decomposed in the Mg–Al alloy, and the solid-soluted Ca atoms diffused along the α-Mg grain boundaries. Al atoms also diffused to the grain boundaries because of attraction to the Ca atoms resulting from a strong reactivity between Al and Ca. As a result, needle-like (Mg,Al)_2_Ca intermetallics were formed as intermediate precipitates in the initial reaction stage during the heat treatment. Finally, the precipitates were transformed into spherical Al_2_Ca particles by the substitution of Al atoms for Mg atoms in (Mg,Al)_2_Ca after a long heat treatment.

## 1. Introduction

Magnesium alloys are remarkably lightweight due to the low density of Mg (~1.74 g/cm^3^). Their application to structural components in automobiles can improve fuel consumption [1,2,3,4]. In particular, the weight reduction of engine blocks and transmission cases used at elevated temperatures (120–200 °C) is very important [4,5,6]. However, the tensile strength of Mg alloys drastically decreases at elevated temperatures. This prevents their widespread use in the automotive industry [5,6,7]. Therefore, it is necessary to improve the heat resistance of Mg alloys. Previous studies have reported that the addition of calcium to Mg–Al alloys improved the creep resistance at 200 °C by the formation of Al_2_Ca or (Mg,Al)_2_Ca intermetallic compounds around the α-Mg grain boundaries [8,9,10]. This is because these network-structured compounds prevented both the deformation of α-Mg grains and grain boundary sliding at elevated temperatures. On the other hand, these network-structured compounds potentially cause grain boundary fractures. Fine dispersed particles of intermetallic compounds are more effective to improve the yield stress of Mg alloys via pinning effects.

In the present study, CaO particles are employed as raw materials to disperse fine Al_2_Ca particles in an α-Mg matrix via a solid-state reaction between CaO particles and the Mg–Al alloy. The conventional Mg–Al alloys reinforced with fine Al_2_Ca particles are expected to demonstrate both excellent yield stress by pinning effects and high cost performance because of the use of inexpensive CaO particles as raw materials. However, from a thermodynamic point of view, an Ellingham diagram of the oxide formation [11] indicates that CaO reduction by Mg never occurs in the solid state (up to 650 °C). Therefore, the CaO raw particles should remain in the cast Mg material. In contrast, our previous study [12] reported that CaO reduction with Al_2_Ca and MgO formation occurred in the Mg-6 wt %Al-1 wt %Zn-0.3 wt %Mn (AZ61B) alloy instead of pure Mg because the change in the standard free energy became negative. As a result, Al_2_Ca phases were synthesized in the AZ61B alloy. Furthermore, CaO particles homogeneously dispersed in the α-Mg matrix contributed to forming the Al_2_Ca fine particles via a solid-state synthesis. Another study [13] reported that Mg-6 wt %Al alloy (AM60) powder composites with CaO additive particles demonstrated a higher creep resistance at 175 °C than those of the ADC12 aluminum alloy. This is because these composites were reinforced with Al_2_Ca fine dispersoids formed via a solid-state reaction between the CaO particles and the AM60 alloy.

**Figure 1:**
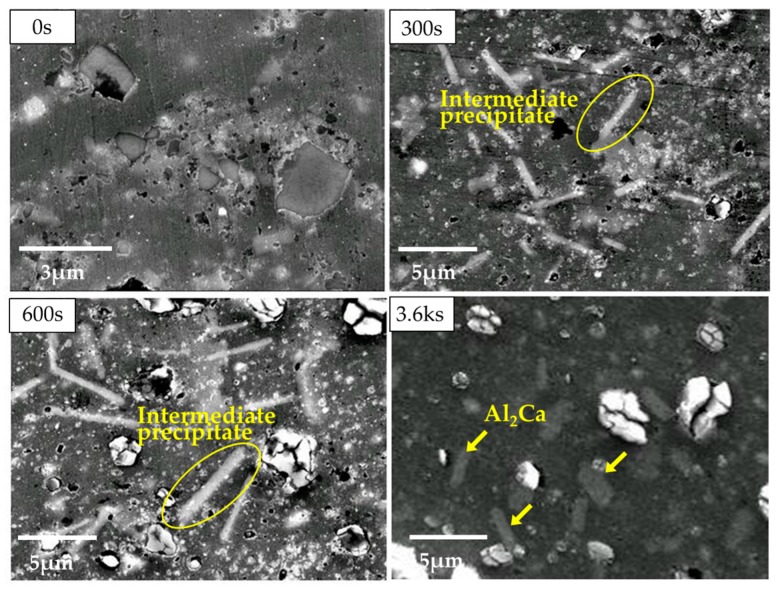
Scanning electron microscopy (SEM) observation on the Mg-Al-CaO precursors with 10-vol % CaO particles after the heat treatment at 500 °C for 0 s, 300 s, 600 s and 3.6 ks.

**Figure 2:**
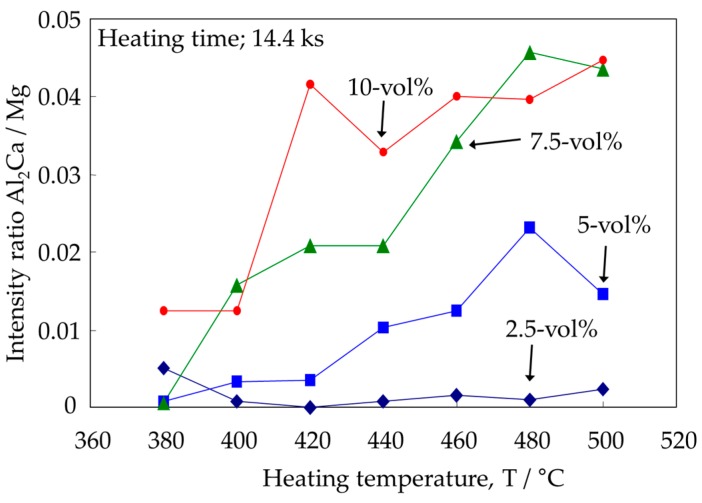
Dependence of X-ray diffraction (XRD) peak intensity ratio of Al_2_Ca and Mg peaks on heating temperature of AZ61B powder precursors with various contents of CaO particles.

Nevertheless, the reaction mechanism for CaO reduction in Mg–Al alloys is not yet clear. For example, a previous study [12] revealed that needle-like intermetallics were formed as intermediate precipitates from the solid-state reaction, as shown in Figure 1. However, the crystal structure was not completely identified. Furthermore, the reaction process of the Al_2_Ca synthesis was also not clear. The same study [12] suggests that the progress of this reaction is governed by the diffusion behavior of Al atoms to CaO particles. This is because Al_2_Ca formation was more clearly detected after heat treatment at higher temperatures as shown in Figure 2 [12]. By contrast, Figure 2 also reveals that the progress of the Al_2_Ca synthesis was promoted by the increase of CaO additive particles. In particular, no Al_2_Ca was detected in the Mg–Al–CaO precursor with 2.5-vol % CaO particles. This reason cannot be well-explained by only the diffusion behavior of Al atoms because all of the CaO particles should contact the Al atoms solid-soluted in the α-Mg matrix during the heat treatment.

In this study, the synthesis mechanism of Al_2_Ca intermetallic compounds via a reaction of CaO additives dispersed in an AZ61B composite precursor during heat treatment was investigated in detail by scanning electron microscopy (SEM), transmission electron microscopy (TEM), electron probe micro-analysis (EPMA), and X-ray diffraction (XRD) analysis. The atomic distribution of Mg, Al, and Ca was determined by TEM-energy-dispersive spectrometry (EDS) and XRD analysis using the AZ61B composite precursor with 10-vol % CaO particles at the initial stage of the heat treatment. The synthesis of (Mg,Al)_2_Ca intermediate precipitates was also detected through crystal structure analysis by XRD and TEM-EDS analysis.

## 2. Materials and Methods

### 2.1. Preparation of AZ61B Precursors Containing CaO Particles

**Figure 3:**
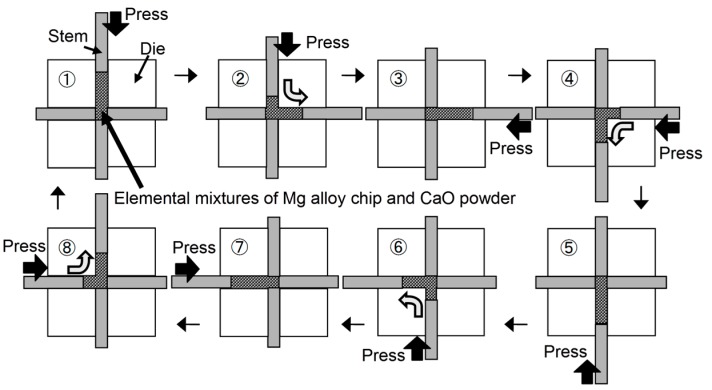
Schematic illustration of one cycle of equal channel angular bulk mechanical alloying (ECABMA) process used in fabrication of Mg alloy powder precursor containing CaO particles.

AZ61B chips machined from their original cast ingot (Al; 6.41, Zn; 1.02, Mn; 0.28, Si; 0.02, Fe; 0.004, Cu; 0.002, Ni; 0.0007, Mg; Bal./mass %) were employed as raw materials. They had a mean particle size of 1.38 mm as measured by a particle size analyzer (LA-950, HORIBA, Kyoto, Japan). CaO particles for use as additives, having a mean particle size of 2.3 μm, were prepared from CaO blocks with a purity of 98% or more via mechanical fragmentation using a ball milling process. The elemental mixtures of the AZ61B chips and CaO particles were mixed by rocking mill equipment (RM-05S, Seiwa Giken Co., Hiroshima, Japan) and used as the starting materials. The content of the CaO additives was 10-vol %. It is effective to disperse CaO additive particles homogeneously in the Mg–Al alloy matrix in order to form Al_2_Ca fine particles via a solid-state synthesis during the heat treatment. Therefore, a severe plastic working was applied to the AZ61B green compacts containing CaO particles by an equal-channel angular bulk mechanical alloying (ECABMA) process [14], where cold compaction and extrusion within a channel bent were alternately carried out in the die, as illustrated in Figure 3. The maximum compaction pressure was 611 MPa. The ECABMA process was repeated 50 times in total resulting in the homogeneous dispersion of the CaO additive particles in the AZ61B alloy chip matrix. This process was also effective for the mechanical breakage of the MgO surface oxide films of the AZ61B chips, and resulted in the formation of newly created Mg active surfaces of the chips that directly came into contact with the CaO particles. The green compact billet (Mg–Al–CaO precursor) prepared by the above process had a 35 mm diameter and an 80 mm length.

### 2.2. Heat Treatment of AZ61B Composite Precursor with CaO Particles

A heat treatment at 500 °C in an argon gas atmosphere was applied to the precursor in order to investigate the reaction behavior between the AZ61B matrix and the CaO particles in the initial stage. The heating time at 500 °C was 300 s. For the investigation of the Al atom distribution in the AZ61B matrix, the aging heat treatment of the AZ61B precursor with no CaO particle was conducted at 200 °C for 86.4 ks in vacuum.

### 2.3. Microstructural Analysis

A field emission scanning electron microscope (JSM-6500F, JEOL, Tokyo, Japan) equipped with an energy-dispersive X-ray spectrometer (EX-64175 JMU, JEOL) was used to investigate the microstructures of the AZ61B powder precursor with no CaO particle. The chemical composition of the Mg–Al matrix was investigated by an electron probe micro-analyzer (EPMA, JXA-8530F, JEOL). The number of the EPMA measurement point was 15, and the mean value was calculated. A transmission electron microscope (JEM-2100F, JEOL) equipped with an energy dispersive X-ray spectrometer (EX-37001, JEOL) was used to investigate the microstructures of the heat treated precursors. X-ray diffraction analysis (XRD-6100, Shimadzu, Kyoto, Japan) was used to clarify the crystal structure of the α-Mg matrix and identify the intermediate precipitates.

## 3. Results and Discussion

### 3.1. Microstructural and Compositional Analysis on the AZ61B Matrix with No CaO Particle

**Figure 4:**
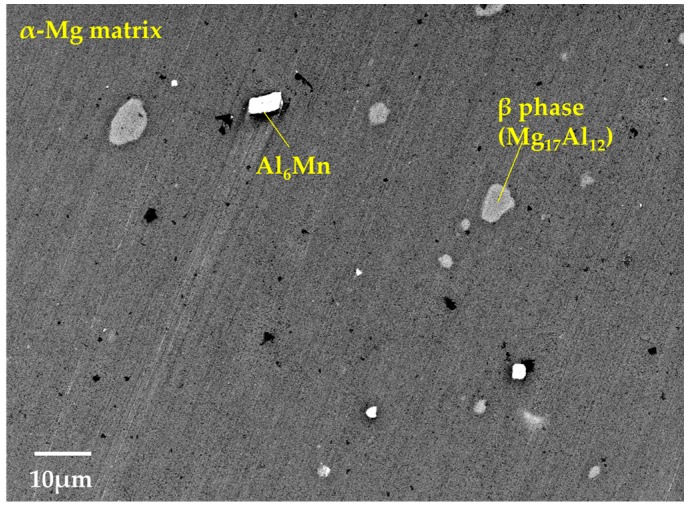
SEM observation on AZ61B powder precursor with no CaO particle after ECABMA process.

**Figure 5:**
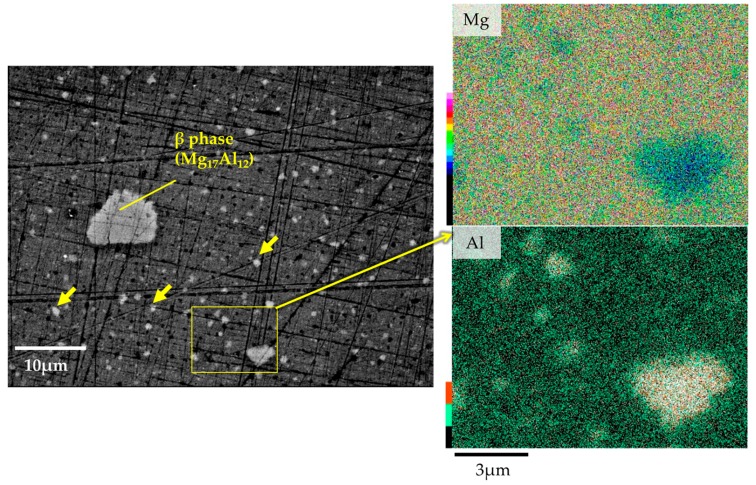
SEM-EDS analysis results of AZ61B powder precursor with no CaO particle after aging heat treatment at 200 °C for 86.4 ks in vacuum.

The microstructures and compositions of the AZ61B powder precursor with no CaO particles were investigated. As shown in Figure 4, SEM on the AZ61B powder precursor after the ECABMA process suggests that coarse β-phases (Mg_17_Al_12_), having a particle size of 2–10 μm, and Al_6_Mn phases were observed in the α-Mg matrix. Table 1 shows the EPMA analysis results for the α-Mg matrix in the precursor. The mean value of the Al composition in the α-Mg matrix was 4.7-at % (5.17-wt %). This means that 80.7% of the entire Al content (6.41-wt %) from the original AZ61B chips was solid-soluted in the α-Mg matrix. The remaining 19.3% contributed to the formation of β-phases and/or Al_6_Mn compounds. The Mg–Al binary phase diagram [15] suggests that a solid solubility limit of Al into the α-Mg phase is 1-at % or lower below 100 °C. Therefore, the above 4.7-at % Al elements existed as a supersaturated solid-solution state in the matrix of the AZ61B precursor. Figure 5 shows the SEM-EDS analysis results of the precursor after the aging heat treatment at 200 °C for 86.4 ks in vacuum. Fine β-phases of less than ~1 μm homogeneously existed as dispersoids in the α-Mg matrix as well as the coarse β-phases. This means that the α-Mg matrix of the precursor contained an excess of Al atoms. Fewer Al atoms could be solid-soluted in the α-Mg phase with a thermal stability below 200 °C.

### 3.2. Microstructural Analysis on the α-Mg Phase of Mg–Al–CaO Precursors

**Figure 6:**
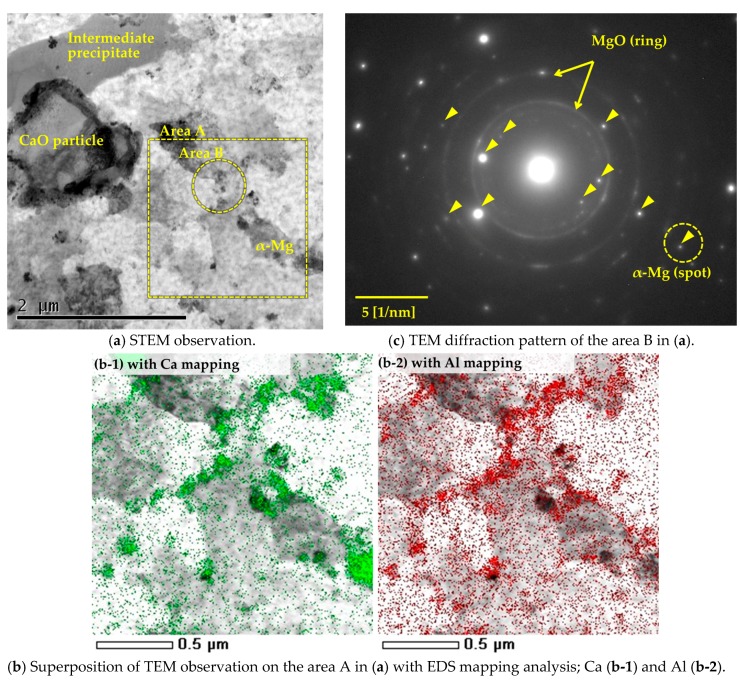
Transmission electron microscopy (TEM) observation result of the Mg-Al-CaO precursor with 10-vol % CaO particles after the heat treatment at 500 °C for 300 s. STEM: scanning transmission electron microscopy; EDS: energy-dispersive spectroscopy.

Microstructural changes of the AZ61B composite precursor with 10-vol % CaO particles at the initial reaction stage were investigated by applying a heat treatment at 500 °C for 300 s. As shown in Figure 6a, the original CaO particles and in situ-formed intermediate precipitates observed using scanning transmission electron microscopy (STEM) were dispersed in the α-Mg polycrystalline matrix. Figure 6b shows a superposition of the TEM observation with an EDS mapping analysis on the α-Mg phase in the same sample at area A. The distributions of the Ca and Al elements were almost the same. Most elements were detected along the α-Mg grain boundaries. Figure 6c shows an electron diffraction pattern for the α-Mg phase at area B. Numerous MgO spots were detected. This is because the AZ61B chips had the original natural oxide surface films, and the additional oxidation of the AZ61B powder precursor occurred during the ECABMA process. Except for MgO and the α-Mg matrix, no other compound was detected. According to these results, it was concluded that Al and Ca atoms in Figure 6b were solid-soluted in the α-Mg matrix with no formation of Al–Ca compounds.

**Figure 7:**
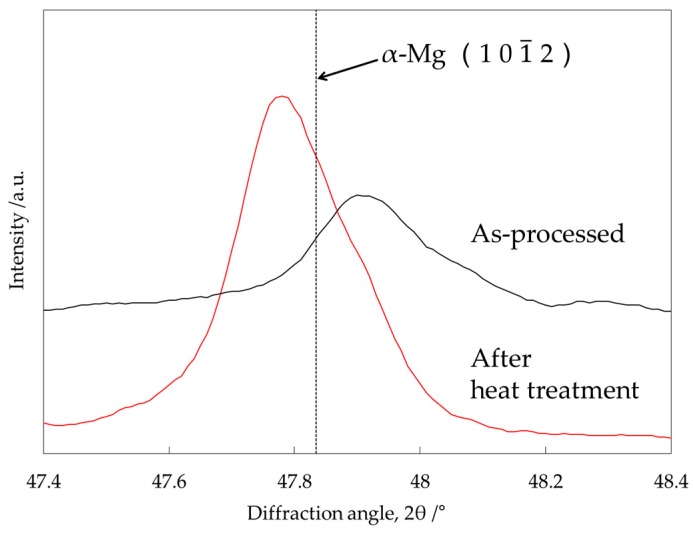
XRD patterns of the Mg–Al–CaO precursors with 10-vol % CaO particles in the as-processed state and after the heat treatment at 500 °C for 300 s.

**Figure 8:**
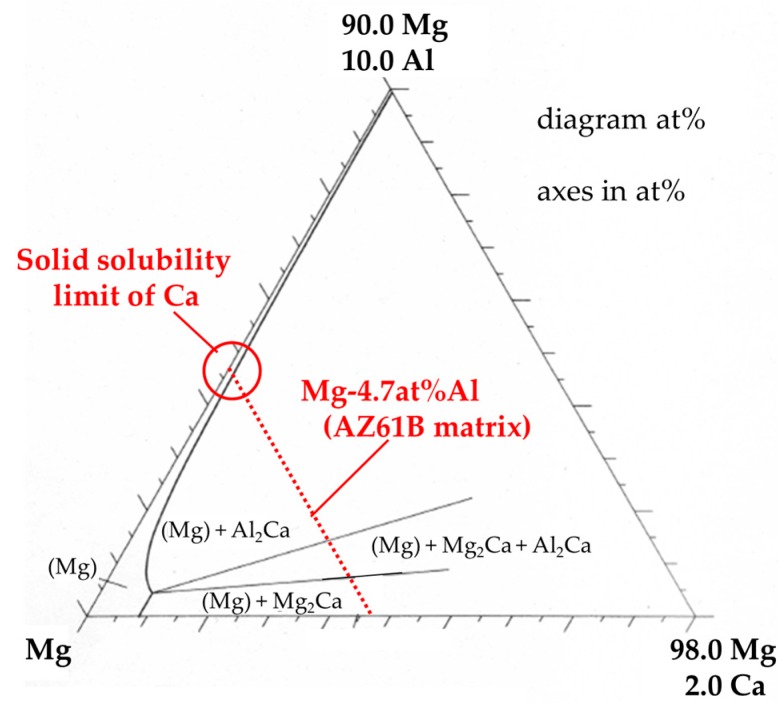
Isothermal section of the Mg–Al–Ca ternary system in Mg-rich corner at 450 °C.

XRD analysis on the same precursor in the as-processed state (before heat treatment) and after the heat treatment at 500 °C for 300 s was carried out in order to investigate the crystal structure of α-Mg by solid solution. As shown in Figure 7, the XRD peak of the α-Mg phase in the as-processed state shifted to a higher angle compared to the original peak illustrated by the dotted line (2θ = 47.83°). This shift occurs because most of the Al atoms existed as a supersaturated solid-solution state in the α-Mg matrix of the as-processed precursor before heat treatment as mentioned in the previous section. Solid-soluted Al atoms created a smaller lattice spacing in α-Mg because the atomic radius of Al (1.43 Å) is smaller than that of Mg (1.60 Å) [16]. On the other hand, the same peak after the heat treatment at 500 °C for 300 s shifted to a lower angle. This is because the solid-soluted Al atoms in the α-Mg matrix decreased by forming intermediate precipitates containing Al and Ca as shown in Figure 6a and the previous study [12]. In addition, the XRD peak shifted to an even lower angle than the original α-Mg peak, as shown by the dotted line. This means that Ca atoms were also solid-soluted in the α-Mg matrix. The lattice spacing became larger than that of the original α-Mg because of the larger atomic radius of Ca (1.97 Å) compared to Mg (1.60 Å). Figure 8 shows an isothermal section of the Mg–Al–Ca ternary system in the Mg-rich corner at 450 °C [17]. It suggests that only a few of the Ca atoms are soluted in the α-Mg matrix, where the Al content is 4.7-at % as shown in Table 1. As a result, Ca atoms can be solid-soluted with thermal stability without Al–Ca precipitate formation when the Ca composition in the α-Mg matrix is within the solid solubility limit.

According to the above consideration, when the heat treatment at 500 °C was applied to the Mg–Al–CaO precursor, CaO particles were thermally decomposed and Ca atoms were solid-soluted in the α-Mg matrix. Then, Ca atoms diffused along α-Mg grain boundaries as shown in Figure 6(b-1). Al atoms originally solid-soluted in the α-Mg matrix also diffused to the grain boundaries as displayed in Figure 6(b-2). This is because Al atoms were attracted to the Ca atoms due to a strong combination of Al and Ca elements [18]. As a result, the needle-like intermediate precipitates containing Al and Ca atoms were formed along the grain boundaries as shown in Figure 1.

**Figure 9:**
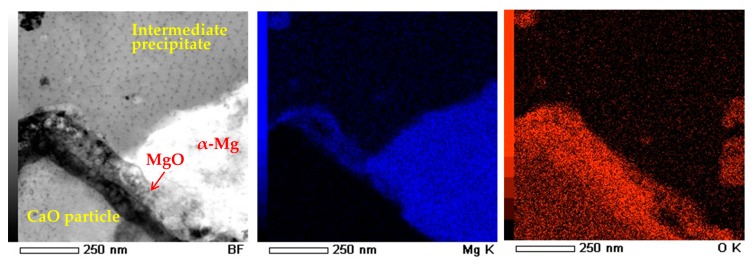
TEM-energy-dispersive spectroscopy (TEM-EDS) mapping analysis around CaO particle in the Mg–Al–CaO precursor with 10-vol % CaO particles after the heat treatment at 500 °C for 300 s.

Figure 9 shows TEM-EDS mapping analysis around a CaO particle of the precursor after the heat treatment at 500 °C for 300 s. MgO thin layers were observed around the CaO particle. They were formed by the solid-state reaction between oxygen atoms originated from CaO and Mg atoms in the α-Mg matrix. This suggests that both CaO reduction and Mg oxidation occurred at the interfacial surface between CaO particles and the α-Mg matrix. In conclusion, the following reaction occurred in the precursor:

Mg–Al solid solution + CaO → Mg–Al–Ca solid solution + MgO.(1)

Based on the above consideration, it is expected that CaO reduction could occur in the Mg–Al alloy regardless of CaO content. However, our previous study [12] reported that no formation of an Al_2_Ca phase was detected in a Mg–Al–CaO precursor with 2.5-vol % CaO particles as shown in Figure 2. This is possibly because a lower CaO content caused a smaller amount of Ca atoms to be solid-soluted in the matrix. This reaction behavior must be investigated in detail for situations of low CaO content.

### 3.3. Mirostructural Analysis of the Intermediate Precipitates by TEM-EDS

**Figure 10:**
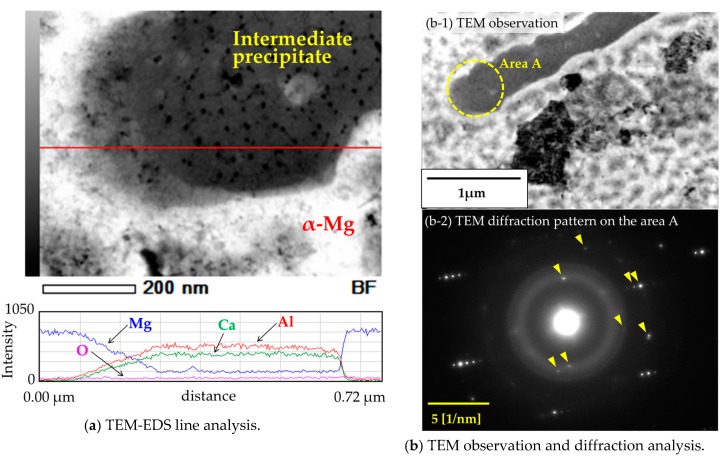
TEM observation on intermediate precipitate in the Mg–Al–CaO precursor with 10-vol % CaO particles after the heat treatment at 500 °C for 300 s.

As previously shown in Figure 1, the spherical Al_2_Ca intermetallic precipitates were formed in the Mg–Al–CaO precursors with 10-vol % CaO particles after the heat treatment at 500 °C for 3.6 ks. On the other hand, a short heat treatment caused the formation of needle-like intermetallics as intermediate precipitates. Our previous study [12] reported that these intermediate compounds were detected as (Mg,Al)_2_Ca or Al_3_Ca_4_Mg. However, the detailed structure was not completely identified. Therefore, a microstructural analysis of the intermediate precipitates was carried out. Figure 10a shows TEM-EDS line analysis results of an intermediate precipitate in the Mg–Al–CaO precursor with 10-vol % CaO particles after the heat treatment at 500 °C for 300 s. Mg, Al, and Ca were detected. Table 2 shows the chemical composition of this precipitate. The (Mg + Al)/Ca ratio was 2.09. Therefore, the intermediate precipitate was identified as (Mg,Al)_2_Ca. Figure 10b shows TEM observation and electron diffraction analysis on the precipitate. Rzychoń et al. [19] reported that a (Mg,Al)_2_Ca Laves phase with a hexagonal C36 structure was formed in the Mg-5Al-3Ca-0.7Sr-0.2Mn alloy. Then, identification of the diffraction pattern in Figure 10(b-2) was determined by referring to the XRD pattern of (Mg,Al)_2_Ca reported in the study [19]. Table 3 shows the referred XRD peak degrees of (Mg,Al)_2_Ca and the lattice spacing calculated by the following Bragg reflection equation:

2d sin θ = nλ(2)

where λ is the X-ray (Cu-Kα) wavelength of 1.5418 Å, n is the diffraction order of 1, and d is the lattice spacing. Diffraction spots indicated by the arrows in Figure 10(b-2) correspond to the lattice spacing of (Mg,Al)_2_Ca according to the data of Table 3. As a result, this compound was identified as a (Mg,Al)_2_Ca phase.

### 3.4. Identification of the Intermediate Precipitates by XRD

**Figure 11:**
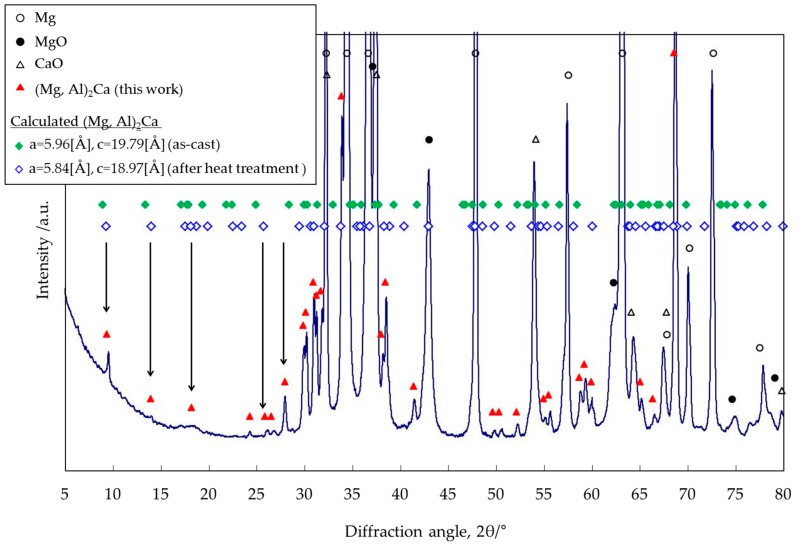
X-ray diffraction pattern of the Mg–Al–CaO precursor with 10-vol % CaO particles after the heat treatment at 500 °C for 300 s.

In the above discussion, a phase identification was carried out by using only the XRD peak degrees (2θ) reported by Rzychoń et al. [19], which were limited within the range from 21° to 66°. In this section, XRD analysis was carried out in order to identify the same intermetallic precipitates by using the lattice spacing calculated from the lattice parameters of the (Mg,Al)_2_Ca phase. Figure 11 shows the XRD profile of the Mg–Al–CaO precursor with 10-vol % CaO particles after the heat treatment at 500 °C for 300 s. The diffraction peaks indicated by triangles (▲) are estimated as the (Mg,Al)_2_Ca compound. The diffraction angles indicated by diamonds (♦ and ◇) were calculated from the lattice spacing of (Mg,Al)_2_Ca in the way described below.

The lattice spacing (dhkl) of the (Mg,Al)_2_Ca phase can be calculated by the following Equation (3) because it has a hexagonal C36 structure:

(3) $$\frac{1}{d_{hkl}}=\sqrt{\frac{4}{3}\frac{h^{2}+hk+k^{2}}{a^{2}}+\frac{l^{2}}{c^{2}}}$$

where *h*, *k*, and *l* are the Miller indices, and a and c are the lattice parameters, respectively. Recently, many studies have reported that four kinds of Laves phases were formed in the Mg–Al–Ca system: Al_2_Ca, Mg_2_Ca, (Mg,Al)_2_Ca, and Al_2_(Mg,Ca) [16,19,20,21,22,23,24]. The lattice parameters of the (Mg,Al)_2_Ca and Al_2_(Mg,Ca) phases have no specific value because of the variable compositions [16,24]. Suzuki et al. [16] reported that the (Mg,Al)_2_Ca Laves phase was crystallized in the Mg–Al–Ca die-cast alloy with a C36 di-hexagonal structure, which was an intermediate between Mg_2_Ca with a C14 hexagonal structure and Al_2_Ca with a C15 cubic structure. According to the previous study [16], in the as-cast state, most of the Al atoms existed in the α-Mg matrix phase as a Mg–Al supersaturated solid-solution. Therefore, the crystal structure and composition of the (Mg,Al)_2_Ca compound were close to the Mg_2_Ca phase. On the other hand, after a heat treatment, both became close to the Al_2_Ca phase because of the Al diffusion and substitution of Al for Mg in the α-Mg matrix contained in (Mg,Al)_2_Ca. Table 4 shows the lattice parameters of the (Mg,Al)_2_Ca compound in the as-cast state and after a heat treatment referred from the previous study [16]. The lattice parameters after the heat treatment became smaller with an increase in the Al concentration of the (Mg,Al)_2_Ca compound. This is because the atomic radius of Al (1.43 Å) is smaller than that of Mg (1.60 Å), as mentioned above.

Based on the above consideration, the lattice spacing of (Mg,Al)_2_Ca was calculated from the two kinds of lattice parameters indicated in Table 4 by using Equation (3). After that, the XRD peak degrees were converted from the lattice spacing by Equation (2). Table 5 shows the calculated results. The Miller indices (*h*, *k* and *l*) were 0, 1, 2, 3, 4, and 5. The calculated values (2θ) are shown within the range from 0° to 80°. The XRD peak degrees calculated from both lattice parameters in Table 4 were similar. These values after the heat treatment were a little larger than those in the as-cast state due to the smaller lattice parameter. The calculated diffraction angles in the as-cast state and after the heat treatment are indicated by two kinds of diamonds (♦ and ◇), respectively, shown in Figure 11. Not all calculated peaks of the (Mg,Al)_2_Ca Laves phase were detected because the calculated results in Table 5 included no information about their intensities. The diffraction peaks estimated as (Mg,Al)_2_Ca (▲) roughly correspond to those of the calculated ones (♦ and ◇). The low-angled peaks indicated by arrows matched them especially well. As a result, the intermediate compound was identified as the (Mg,Al)_2_Ca Laves phase. In addition, the detected peaks at ~9.5° and ~14° correspond to the calculated ones for after the heat treatment rather than those of the as-cast state. According to the above analysis and discussion, this suggests that the crystal structure and compositions of the intermediate compound are closer to Al_2_Ca than they are to Mg_2_Ca. This also agrees with the result showing that the Al content of the compound was much higher than the Mg content as shown in Table 2. It is expected that the crystal structure of the intermediate precipitate becomes much closer to that of Al_2_Ca when a long heat treatment is employed. This is because Al atoms in the α-Mg matrix are substituted for Mg atoms contained in (Mg,Al)_2_Ca. As a result, Al_2_Ca compounds were homogeneously dispersed after the heat treatment for 3.6 ks as shown in Figure 1. Their shapes became spherical in order to reduce the surface energy.

## 4. Conclusions

AZ61B composite powder precursors with 10-vol % CaO additive particles prepared via the ECABMA process were heat treated to synthesize Al_2_Ca intermetallic precipitates in a solid-state reaction. SEM-EDS and EPMA were applied to investigate the distribution of Al atoms in the AZ61B powder precursor. The precursor before the heat treatment indicated that the α-Mg matrix contained 4.7-at % Al atoms and the remaining Al elements contributed to forming Mg_17_Al_12_ or Al_6_Mn compounds in the matrix. TEM-EDS and XRD analysis on the AZ61B composite precursor in the initial reaction stage showed that CaO additives were thermally decomposed in the Mg–Al alloy matrix, and solid-soluted Ca atoms diffused along the α-Mg grain boundaries. Al atoms also diffused to the grain boundaries because of their strong affinity for Ca atoms. As a result, needle-like (Mg,Al)_2_Ca intermetallics were formed as intermediate precipitates at the initial stage of the heat treatment. They were completely transformed into the spherical Al_2_Ca phases by substitution of Al atoms for Mg atoms of the above (Mg,Al)_2_Ca intermediates after a long heat treatment.

## Figures and Tables

**Table 1:** Chemical compositions of the α-Mg matrix in AZ61B powder precursor with no CaO particle.

Element (at %)	Al	O	Zn	Mn	Mg	Total
α-Mg matrix	4.70	0.27	0.26	0.03	94.74	100.00

**Table 2:** Chemical compositions of intermediate precipitates in the Mg–Al–CaO precursor with 10-vol % CaO particles after the heat treatment at 500 °C for 300 s.

Element (at %)	Mg	Al	Ca	O	Mn	Zn	Total
Intermediate precipitate	12.34	48.84	29.29	8.98	0.16	0.40	100.00

**Table 3:** (Mg,Al)_2_Ca peak degrees of X-ray diffraction in Rzychoń’s work [19] and calculated lattice spacing from these peak degrees.

XRD Peak Degree, 2θ (°)	Calculated Lattice Spacing, d (Å)
29.44	3.033
30.65	2.916
33.53	2.673
38.20	2.356
43.01	2.103
49.54	1.840
56.80	1.621
61.57	1.506

**Table 4:** Crystal structures and lattice parameters of (Mg,Al)_2_Ca Laves phases [16].

Compound	Type	Structure	Lattice Parameter	
			a (Å)	c (Å)
(Mg,Al)_2_Ca	C36	di-hexagonal (as-cast)	5.96	19.79
		di-hexagonal (after heat treatment)	5.84	18.97

**Table 5:** Miller indices and calculated diffraction angles of (Mg,Al)_2_Ca Laves phase.

Miller Indice				Diffraction Angle, 2θ (°)		Miller Indice				Diffraction Angle, 2θ (°)	
*h*	*k*	*i*	*l*	As-Cast (a = 5.96(Å)) (c = 19.79(Å))	After Heat Treatment (a = 5.84(Å)) (c = 18.97(Å))	*h*	*k*	*i*	*l*	As-Cast (a = 5.96(Å)) (c = 19.79(Å))	After Heat Treatment (a = 5.84(Å)) (c = 18.97(Å))
0	0	0	1	4.46	4.66	2	1	−3	4	50.27	51.55
0	0	0	2	8.94	9.32	2	1	−3	5	52.28	53.69
0	0	0	3	13.42	14.01	3	0	−3	0	53.24	54.42
1	0	−1	0	17.18	17.53	3	0	−3	1	53.46	54.65
1	0	−1	1	17.76	18.15	3	0	−3	2	54.10	55.35
0	0	0	4	17.93	18.71	3	0	−3	3	55.17	56.49
1	0	−1	2	19.40	19.89	3	0	−3	4	56.64	58.07
1	0	−1	3	21.86	22.51	3	0	−3	5	58.49	60.05
0	0	0	5	22.46	23.45	2	2	−4	0	62.31	63.74
1	0	−1	4	24.93	25.75	2	2	−4	1	62.51	63.95
1	0	−1	5	28.42	29.43	2	2	−4	2	63.10	64.58
1	1	−2	0	29.98	30.62	2	2	−4	3	64.07	65.63
1	1	−2	1	30.33	30.99	3	1	−4	0	65.16	66.68
1	1	−2	2	31.35	32.07	3	1	−4	1	65.36	66.88
1	1	−2	3	32.98	33.81	2	2	−4	4	65.41	67.07
2	0	−2	0	34.76	35.50	3	1	−4	2	65.93	67.50
2	0	−2	1	35.06	35.82	3	1	−4	3	66.88	68.52
1	1	−2	4	35.15	36.12	2	2	−4	5	67.12	68.91
2	0	−2	2	35.96	36.78	3	1	−4	4	68.19	69.93
2	0	−2	3	37.42	38.33	3	1	−4	5	69.87	71.74
1	1	−2	5	37.79	38.92	4	0	−4	0	73.37	75.14
2	0	−2	4	39.38	40.42	4	0	−4	1	73.55	75.33
2	0	−2	5	41.78	42.98	4	0	−4	2	74.10	75.92
2	1	−3	0	46.55	47.57	4	0	−4	3	75.00	76.89
2	1	−3	1	46.79	47.82	4	0	−4	4	76.25	78.25
2	1	−3	2	47.50	48.58	4	0	−4	5	77.86	79.98
2	1	−3	3	48.67	49.83	-	-	-	-	-	-
